# Transoesophageal Echocardiography for Monitoring Liver Surgery: Data from a Pilot Study

**DOI:** 10.1155/2012/723418

**Published:** 2012-04-30

**Authors:** Filipe Pissarra, Antonio Oliveira, Paulo Marcelino

**Affiliations:** ^1^Anesthesiology Department, Hospital Curry Cabral, Rua da Beneficência 8, 1069-166 Lisbon, Portugal; ^2^CEDOC, Faculdade de Ciências Médicas, Lisbon, Portugal

## Abstract

A pilot study aimed to introduce intraoperative monitoring of liver surgery using transoesophageal echocardiography (TEE) is described. A set of TEE measurements was established as a protocol, consisting of left atrial (LA) dimension at the aortic valve plane; mitral velocity flow integral, calculation of stroke volume and cardiac output (CO); mitral annular plane systolic excursion; finally, right atrial area. A total of 165 measurements (on 21 patients) were performed, 31 occurring during hypotension. The conclusions reached were during acute blood loss LA dimension changed earlier than CVP, and, in one patient, a dynamic left ventricular (LV) obstruction was observed; in 3 patients a transient LV systolic dysfunction was documented. The comparison between 39 CO paired measurements obtained by TEE and PiCCO2 revealed a statistically significant correlation (*P* < 0.001, *r* = 0.83). In this pilot study TEE successfully answered the questions raised by the anesthesiologists. Larger cohort studies are needed to address this issue.

## 1. Introduction

In major surgery haemodynamic complications are likely to occur; hence for this reason monitoring is necessary to trace physiological parameters. There are several commercially available monitoring systems, but transoesophageal echocardiography (TOE) was not as extensively studied in noncardiac [1] as it was in cardiac surgery [2–5]. Due to its unique ability for cardiac imaging, assessing left ventricular (LV) function and right heart chambers dimensions, it is considered promising [5].

 The questions faced by anaesthesiologists in noncardiac surgery are quite different from those in cardiac surgery, where valvular diseases, prosthesis placement and complications are the most relevant. Questions on LV function or acute change in volume status and hypotension are more concerning in noncardiac surgery. Good candidates for such monitoring are patients submitted to major surgery, especially those undergoing liver surgery or even transplantation [6–8]. During this type of surgery, haemodynamic instability can occur during liver manipulation or due to associated blood loss.

In our centre, the usual means for monitoring include the continuous monitoring of the central venous pressure (CVP) and, in selected cases, the continuous monitoring of cardiac output (CO) through the use of the PiCCO system. Pulmonary artery catheters, used more often in the past, are now seldom used. As resident anaesthesiologists felt an increasing need for a more accurate monitoring, a pilot study aimed to introduce intraoperative monitoring of liver surgery using TOE was performed. This study was aimed to evaluate the place of TOE for liver surgery monitoring and to compare efficiency of TOE measurements with PVC and PiCCO to diagnose hemodynamic instability causes. A set of TOE measurements was established as a protocol, after previous discussion with the anaesthesiology staff about the required information. A comparison between the information derived from the monitoring devices used was also performed.

## 2. Material and Methods

### 2.1. Patients

This was a 1-year prospective study, which included patients submitted to liver surgery and enrolled without previous selection, although limited to the availability of the anesthesiologists (FP and AO), intensive care specialist with expertise in the area (PM), and echocardiography equipment. This pilot study was open with the anaesthesiologists being aware of TOE information. All data was digitally recorded for later visualisation, if deemed necessary.

Patients were characterized by age, gender, and body surface area. Main diagnoses (for surgical purposes) and comorbidities were also collected. The main demographic and clinical characteristics of the enrolled patients are presented in Table 1.

The study protocol was reviewed by the local Ethics Board, and an informed statement was obtained previous to surgery.

### 2.2. Methods

During liver surgery, hypotension and liver manipulation (reported by the surgeons) were the most regarded situations. Hypotension was considered when mean arterial blood pressure was 60 mmHg or lower, and data was thoroughly analysed. Blood loss was considered either by the reports from the surgeons or by a decrease in haemoglobin levels of more than 2 gr/dL. Other possible aetiologies were evaluated according to the available monitoring devices.

Patients were anaesthetised using a general balanced anaesthesia, having been intubated after anaesthesia induction.

CVP monitoring was performed continuously using a central venous line connected to a Philips M4 monitor, where the arterial pressure and heart rate were also registered. The arterial pressure was monitored invasively using an arterial catheter inserted into a radial or femoral artery. The invasive CO, when used, was determined using a PiCCO 2 system, for which a central venous line and a femoral arterial line were inserted and then calibrated according to the manufacturer's instructions.

### 2.3. Echocardiography

The TOE monitoring was performed using a Siemens ACCUSON X300 and a General Electric LOGIC P6, both equipped with a multiplane transoesophageal probe.

Before the study started, a consensus was established with the anaesthesiologists to determine the information needed for monitoring. The information considered necessary was previous surgery knowledge of the heart anatomy and function; CO; left ventricular (LV) performance; data on volume status; right heart chamber evaluation. Special concern was addressed to the TOE parameters; they needed to be easily obtained, not time consuming, in order to permit quick therapeutic decisions. It was also established that intragastric views should not be used so as to avoid interference with the surgical field. The choice of invasive monitoring was carried out by the anaesthesiologist's judgement and independent of study purposes.

**Figure 1:**
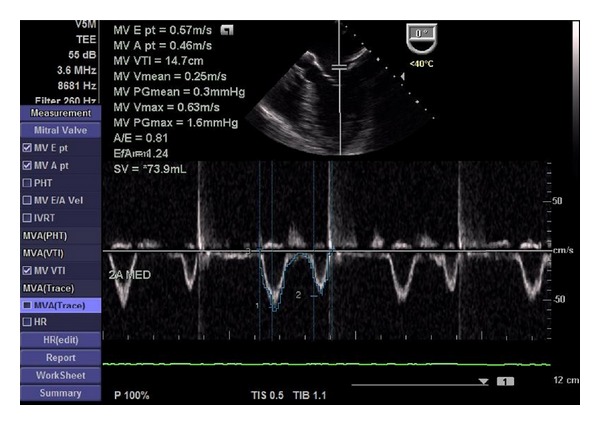
Determination of the mitral VTI.

**Figure 2:**
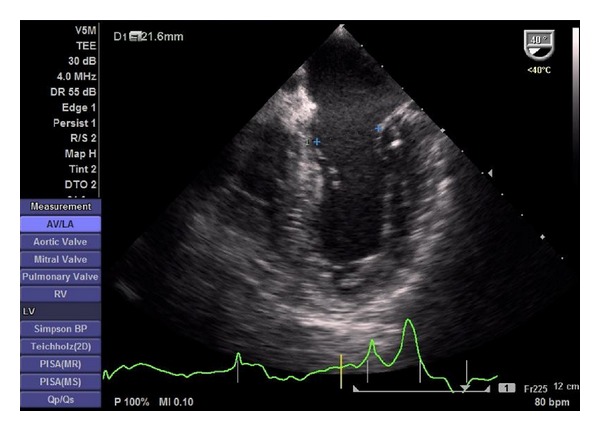
Determination of the mitral annulus diameter.

**Figure 3:**
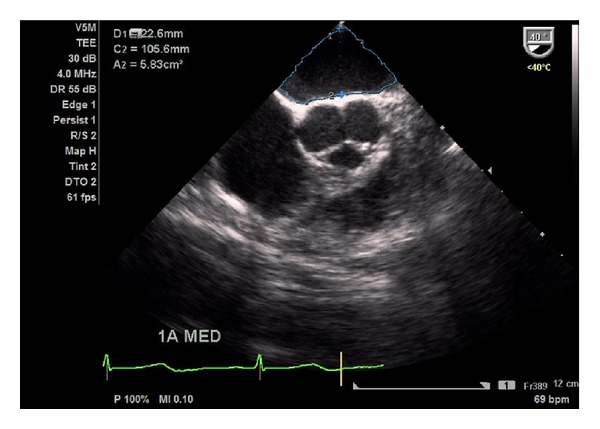
Determination of visible LA area and dimension (distance from the first echo from LA to aortic valve) in the aortic plane.

**Figure 4:**
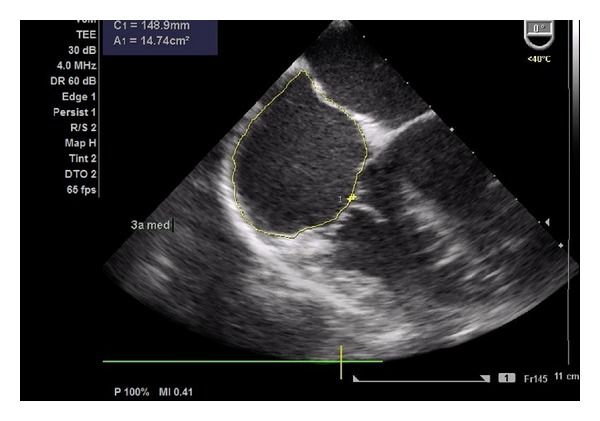
Determination of the right atrial area.

After anaesthesia induction, a transoesophageal probe was inserted and the first images obtained. A global examination was first performed and global and segmental wall motion abnormalities were evaluated, as well as valvular regurgitations. The following sets of measurements were chosen in order to obtain the information previously required by the anaesthesiologists. The CO was obtained through the mitral velocity time integral (VTI), measured as follows. First the left ventricular influx by evaluation of the mitral E/A ratio in the 4-chamber view was analysed. Secondly, left ventricular CO was assessed by measuring the mitral VTI, calculating the stroke volume index (SVI) and multiplying it by heart rate (Figure 1). Necessary information with regards to the width of the mitral valve orifice was measured in the same view (Figure 2). The LV function was assessed through the external mitral annulus systolic excursion (MAPSE, considered the most feasible parameter compared to ejection fraction and other volumetric parameters) obtained in the same 4-chamber view. At the aortic valve plane, during diastole when the three aortic cuspids were visible, visible left atrium (LA) area and dimension, obtained from the LA first echo to aortic valve (Figure 3), were determined. Lastly, the assessment of right heart chambers was performed; the probe was repositioned for the assessment of the right atrium and ventricle. The measurement of the right atrial area was emphasized (Figure 4). All TOE measurements were performed at end-expiration, and other changes detected during TOE were registered. TOE evaluation was performed routinely every 15 minutes of surgery or whenever considered necessary if hypotension, blood loss, or liver manipulation were reported.

An LV systolic dysfunction was considered whenever MAPSE was <15 mm, and CO <2,4 L/m^2^. LA and RA dimensions were considered the TOE surrogates for volume status and preload determination and regarded as changes from the previous measurements.

### 2.4. Statistical Analysis

All variables are presented as mean and standard deviations. To compare continuous variables, parametric and nonparametric statistical tests were used, calculating the correlation index (*r*) and *P* value, which were considered significant if <0.05. The statistical program used was an SPSS for Windows, version 18.0 (SPSS Inc, Chicago, Illinois). Comparisons were made between data from TOE and usual monitored parameters. The parameters compared were CO analysis by PiCCO and TOE whenever available and preload assessment comparing CVP with LA and RA measurements, as well as its changes during acute phenomena.

## 3. Results

Overall, 165 TEE dataset measurements were performed, and in 5 patients a PiCCO2 system was present. Overall, 31 registries were performed during hypotension. Of these, 16 (5 patients) were due to hemorrhage, 9 (5 patients) without obvious cause, and 6 (4 patients) due to liver manipulation.

**Figure 5:**
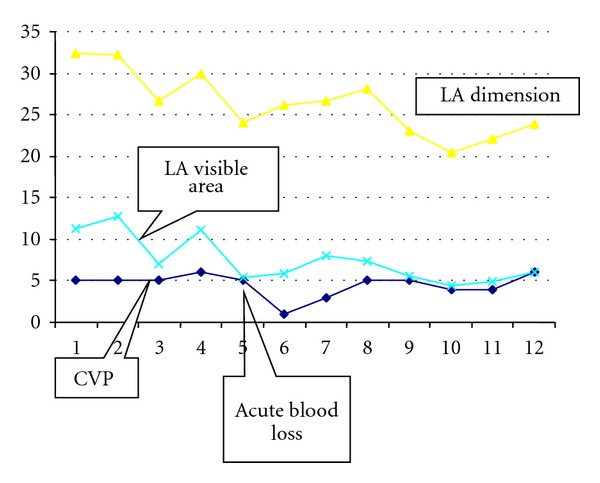
Line graphic of a patient with an episode of acute blood loss, comparing the time of CVP, LA dimension, and visible LA area changes. Note that the LA parameters changed earlier than CVP.

 In the haemorrhage evaluation the LA and RA dimensions decreased in all patients, as well as CVP, but it occurred simultaneously in only two occasions. In the remaining measurements (*n* = 29), TOE modifications preceded CVP changes by 10 to 15 minutes. In Table 2, and in Figure 5, a graphic representing a registry during an acute blood loss and changes in CVP and visible LA area and dimension is presented. It was also observed that the LA dimension decreased almost uniformly by nearly 20% (19.8%  ±  0.9). The comparative data of the parameters previous to haemorrhage and during haemorrhage is presented.

There were 6 cases of liver manipulation. In two episodes hypotension occurred without changes in CVP. Interestingly, LA and RA dimensions decreased during liver manipulation, but CVP and CO remained unchanged. The comparative data obtained previous to and during liver manipulation are presented in Table 3.

In 9 cases (5 patients), a hypotensive episode was documented without blood loss. Within this group, in two cases a typical change in volume status was detected by TOE, but not by CVP; in one case, a decreased volume status was identified by both methods; in two cases there was no change observed by the two methods; in three episodes (3 patients) a systolic dysfunction was detected by TOE (decrease in CO and MAPSE) in patients with previous normal LV contractility. This LV dysfunction was transient and, due to global LV hypokinesia, the recovery was observed within a few minutes. No apparent cause for this phenomenon was detected.

Only one patient presented an LV dysfunction, detected previous to surgery, suffering from ischemic heart disease. During surgery, hypotension was detected during a massive blood loss, and LV dysfunction exacerbated, along with exacerbated wall motion abnormalities. Vasopressor and inotropic support was started, some recovery of LV contractility was observed but the patient remained hypotensive. This patient died in the early postoperative period in the Intensive Care Unit.

Overall, TOE-derived CO varied more markedly than PiCCO2-derived; the mitral E/A wave form changed during anaesthesia induction and remained less than one during most part of the surgery. The first obtained mean values for this parameter were 0.99 ± 0.47 and for the remaining 0.83 ± 0.36 (*P* = 0.001). However no relevant information could be obtained from this parameter during surgery, even during hypotension/blood losses.

By linear regression analysis, considering CVP as a dependent variable and LA dimension and RA area as independent variables, a significant association was found between CVP and RA area (*P* = 0.001), but not between CVP and LA dimension (*P* = 0.07).

**Figure 6:**
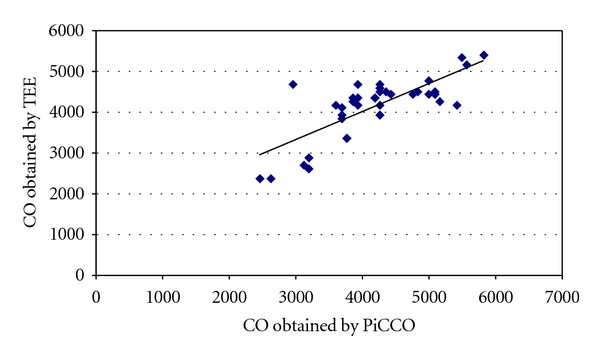
Dispersion graphic comparing the cardiac output obtained by PiCCO2 and TEE (*P* < 0.001, *r* = 0.83).

In 5 patients a comparison of the CO by TEE and PiCCO was possible, consisting in 39 paired simultaneous measurements. In Figure 6 the linear correlation is presented; the *P* value is <0.001, and the correlation coefficient was 0.83 (Figure 6). The mean error between CO obtained by TOE and PiCCO2 was 63.6 ± 528.2 (limits: −1722 to 1230).

## 4. Discussion

Data from this pilot study highlighted several possibilities of TOE as an intraoperative monitoring tool for liver surgery. It also brings some new data that would be subjected to further studies and analyses. The hypotension episodes observed tested the clinical utility of TOE monitoring. It could effectively detect changes in CO and detected changes in volume status earlier than comparative pressure-derived methods, along with the LV function monitoring.

The assessment of volume status is a major concern for any surgery. CVP monitoring was the “standard” method used. The comparative analysis of invasive and noninvasive parameters revealed some new data. In most cases of acute volume loss, as seen in major haemorrhage, LA dimensions changes were observed earlier, by 10 to 15 minutes, which may be considered an early adaptive phenomenon in order to ensure LV filling pressure (during volume loss the decrease in LA dimension prevents further decrease in LA pressure and consequent LV filling pressure). Interestingly, the same changes were observed during liver manipulation, which results in decreased preload due to vascular compression. This information is important and allows the anaesthesiologist to anticipate adequate therapeutic actions. To our knowledge, this finding has not yet been described in the literature. However, RA area was the parameter with a statistically significant association with CVP, not the LA dimension.

The present study evaluated preload not preload dependency, using comparative data from static parameters. Among the possible parameters, left ventricular end diastolic area (LVEDA) could not be considered as transgastric views were not obtained [9]. Dynamic concepts for fluid administration [10, 11] and preload dependency were not also considered in the present study. Only when PiCCO system was inserted could the anaesthesiologists evaluate the systolic volume variation, and fluids were often administered whenever this parameter was >15%, regardless of haemodynamic status. Several TOE parameters can be used to assess preload dependency [12, 13], and in some settings they were used to guide intraoperative fluid administration. In this regard we must consider that the protocol was formulated in order to detect and characterize acute changes, not to guide fluid administration. The emphasis was acute volume loss mainly blood losses that should be rapidly treated. In other words, we focused on acute phenomena.

CO has gained particular attention as a way of accessing the global circulatory status, but how accurately this variable measures the adequacy of circulatory flow is yet to be established. Perhaps the usefulness of CO consists in detecting changes in this variable during surgery, especially during episodes of instability. Considering this as the main use of CO monitoring, the changes are more important than its absolute value. Using TEE, CO was monitored through the mitral pulsed-Doppler influx, an occasionally used method [14, 15]. In this method mitral valve annulus was used as a surrogate for cross-sectional area. The accuracy of mitral valve stroke volume is debatable. The mitral valve orifice does not have a perfect geometrical shape; thus it is not used by investigators. As we decided not to use intragastric views in order not to interfere with surgery, this was the possible, non-time-consuming method. The correlation obtained with the PiCCO system was statistically significant (*P* < 0.001), with *r* value of 0.83. Although the methods are different the importance of this parameter is its changes during acute events, and in this regard both methods were reliable, although TOE-derived CO presented greater variability than PiCCO-derived CO.

Left ventricular function was monitored through mitral valve annular plane systolic excursion, a method widely used and tested [16, 17]. The LV function monitoring ability is perhaps one of the most important features of TOE monitoring. No other means is comparable not even the classic methods. It was a valuable tool in the approach of hypotension in one patient, guiding inotropic and vasopressor support and detecting a transient LV dysfunction in other 3 episodes of hypotension. This detection was only possible because TOE monitoring was present, and we could not detect a cause for this phenomenon. Also, we could not find a similar description in the literature. Although an experienced observer could detect changes in LV function subjectively, MAPSE was used in this pilot study as an objective measurement. One should remember that LV systolic dysfunction can also be easily detected by simultaneous changes in mitral VTI and MAPSE.

Other possibilities of TOE were not observed in this study, for example, the detection of right heart overload and alterations in cardiac chambers, mainly due to gas embolism or thrombus formation. In a larger cohort study they could possibly be observed.

## 5. Study Limitations

In this pilot study the preload determination was considered rather than preload dependency. The invasive counterpart for preload dependency estimation can be the systolic volume variation, and several TOE parameters can be used to evaluate, such as the analysis of superior vena cava, an easy procedure to carry out during TOE examination. This need was not particularly expressed by anaesthesiologists, more focused on acute and life-threatening phenomena and LV function. But in future protocols this item can be used. Some other measurements could be considered but, as we limited the information to a non-time-consuming acquisition in order to describe an easy-to-use tool during anaesthesia, most information was limited. More complex data can be obtained through this technique which, yet due to time constraints typical of an operating theatre, went beyond the scope of this study.

In the future it is also necessary to enrol patients who present atrial fibrillation, in order to fully understand the limitations of TOE monitoring.

Another question regards TOE possibilities. Right heart dysfunction and/or overload could not be detected in the patients studied, but it can be an advantage in the use of TOE. Other conditions resulting from the cardiac imaging (valvular regurgitations, intracardiac masses or thrombi) can also present an advantage, not observed in the studied patients.

## 6. Conclusion

The use of a TOE monitoring was possible during liver surgery, in order to assess volume status, LV function, and CO. In five patients monitored with the PiCCO system, a statistically significant correlation between CO obtained by mitral valve VTI was obtained. TOE was also useful during episodes of hypotension, detecting changes in volume status earlier than invasive tools.

TOE is a possible and valuable tool in monitoring liver surgery, and its use by anaesthesiologists should be encouraged. More data is needed to establish its role in other noncardiac surgery monitoring.

## Figures and Tables

**Table 1:** Demographic and clinical characterization of studied patients (*n* = 21).

Age (years, mean, and sd)	54.1 ± 17.6
Male (*n*)	12
Body surface area (m^2^, mean and sd)	1.73 ± 0.17
Liver resection due to metastatic disease (*n*)	14
Liver resection due to other diseases (*n*)	4
Liver transplant (*n*)	3
Past history:	
Coronary artery disease	1
Hypertension	2
Diabetes mellitus	2
Other	1

**Table 2:** Comparison of hemodynamic and echocardiographical data in hypotension due to blood losses (16 sets of measurements in 5 patients).

Parameter	Data before hypotension	Data during hypotension	*P*
HR (bpm)	71.7 ± 9.4	74 ± 11.8	ns
CVP (mmHg)	6.7 ± 1.9	5.2 ± 2.2	0.01
LA area (cm^2^)	9.1 ± 3.5	5.4 ± 2.2	0.001
LA dimension (mm)	28.5 ± 4.7	22.8 ± 4.3	0.001
RA area (cm^2^)	15.1 ± 2.4	13.8 ± 2.6	0.01
Mitral E/A	0.73 ± 0.33	0.76 ± 0.34	ns
PiCCO CO (mL/min)	4322 ± 452	3921 ± 404	0.001
TOE CO (L/min)	4571 ± 472	3622 ± 463	<0.001
MAPSE (mm)	16.2 ± 0.9	16.1 ± 1.3	ns

HR: heart rate, bpm: best per minute, CVP: central venous pressure, LA: left atrium, RA: right atrium, TOE: transoesophageal echocardiography, MAPSE: mitral annulus systolic excursion, cm^2^: squared centimetres, mm: millimeters, and mL/min: mililiters per minute.

**Table 3:** Comparison of hemodynamic and echocardiographical data during liver manipulation (6 sets of measurements in 4 patients).

Parameter	Data before liver manipulation	Data during liver manipulation	*P*
HR (bpm)	69 ± 7.6	72.2 ± 9.1	ns
CVP (mmHg)	6.1 ± 1.1	6.1 ± 1.8	ns
LA area (cm^2^)	9.9 ± 3.2	6.2 ± 3.4	0.003
LA dimension (mm)	28.4 ± 3.9	25.2 ± 4.1	0.005
RA area (cm^2^)	15.6 ± 2.2	15.3 ± 2	ns
Mitral E/A	0.77 ± 0.3	0.84 ± 0.39	ns
PiCCO CO (mL/min)	4020 ± 397	4150 ± 425	ns
TEE CO (L/min)	4286 ± 438	4481 ± 467	ns
MAPSE (mm)	16.4 ± 01.1	15.4 ± 1.2	ns

HR: heart rate, bpm: best per minute, CVP: central venous pressure, LA: left atrium, RA: right atrium, TOE: transoesophageal echocardiography, MAPSE: mitral annulus systolic excursion, cm^2^: squared centimetres, mm: millimeters, and mL/min: milliliters per minute.
